# Mesenchymal Stem Cell-Derived Exosomes for Bone Regeneration: Modification Strategies and miRNA-Mediated Modulation of Osteogenic Signaling

**DOI:** 10.3390/ijms27219486

**Published:** 2026-10-23

**Authors:** Anastasiia Meglei, Daria Paymeteva, Irina Nedorubova, Viktoriia Basina, Dmitry Goldshtein, Tatiana Bukharova

**Affiliations:** Research Centre for Medical Genetics, 115478 Moscow, Russia; an.megley95@yandex.ru (A.M.); vika.basina12@gmail.com (V.B.); dvgoldshtein@gmail.com (D.G.); bukharova-rmt@yandex.ru (T.B.)

**Keywords:** mesenchymal stem cells, exosomes, bone tissue regeneration, gene-modified exosomes, BMP2, osteogenesis, immunomodulation

## Abstract

Bone tissue regeneration remains a major clinical challenge, and exosomes secreted by mesenchymal stem cells (MSCs) have emerged as attractive cell-free therapeutic tools. To enhance their efficacy and local action, exosomes can be modified to enrich them with regulatory factors and impregnated into biocompatible matrices. This review summarizes recent advances in engineering MSC-derived exosomes for bone regeneration. Strategies analyzed include physicochemical modification of donor MSCs, direct exosome modification, and genetic modification of MSCs to enrich exosomes with therapeutic molecules such as bone morphogenetic protein 2 (BMP-2), vascular endothelial growth factor (VEGF), and regulatory miRNAs. In addition, the role of these miRNAs in osteogenic signaling was analyzed. Modified exosomes demonstrate enhanced osteogenic, angiogenic, and immunomodulatory effects in vitro and in vivo, particularly when combined with biomaterial scaffolds for sustained release. Thus, engineered MSC-derived exosomes constitute an effective approach for bone tissue engineering and show potential for clinical translation.

## 1. Introduction

Bone tissue regeneration is a complex process dependent on many factors, including cellular and molecular interactions, as well as the influence of a microenvironment. Osteoblasts, osteoclasts and MSCs play a key role in bone repair, jointly regulating remodeling and restoration of damaged areas [1]. Vascularization in the bone defect area ensures the delivery of oxygen, nutrients, and growth factors, while immune cells regulate the inflammatory response, removal of damaged tissues, and activation of regenerative mechanisms [2,3]. Dysregulation of these physiological processes is associated with the pathogenesis of widespread diseases, including osteoporosis, osteoarthritis, and impaired neo-osteogenesis in bone tissue injuries [4]. In recent decades, MSCs have become the basis of bone tissue engineering due to their ability for multilineage differentiation and powerful paracrine activity [5]. However, this approach has a number of limitations, including the risk of immune response, potential tumor formation, low engraftment, and instability of cells after transplantation. In this regard, interest is growing in alternative strategies based on paracrine regulation mediated by the cell secretome. The MSCs secretome is a complex set of soluble factors, including cytokines, chemokines, and growth factors, as well as extracellular vesicles (EVs), which together provide a wide range of paracrine effects [6,7,8]. Extracellular vesicles (EVs) comprise heterogeneous lipid bilayer-delimited particles that can be classified according to their biogenesis and physical characteristics. Exosomes constitute a subtype of small EVs of endosomal origin, whereas other EV subpopulations are commonly described using operational size-based terms, including small EVs (sEVs; typically <200 nm) and medium/large EVs (m/lEVs; typically >200 nm) [9]. Among these populations, small extracellular vesicles (sEVs) attract particular attention. While soluble components of the secretome are directly released into the extracellular space, the membrane structure of vesicles helps protect their contents from the extracellular environment and deliver it to target cells [10,11]. Due to these properties, MSC-derived sEVs are considered a promising component of the secretome and a platform for developing cell-free regenerative approaches [7,12]. The therapeutic efficacy of MSC-derived sEVs in bone tissue regeneration largely depends on the content of the vesicles [10,13]. Native MSC-derived EVs possess basic therapeutic potential: they are able to transfer biologically active molecules (proteins, mRNA, miRNA) that modulate key pro-osteogenic and angiogenic signaling cascades in recipient cells, including the BMP/Smad, Wnt/β-catenin, PI3K/Akt, and NOTCH1/DLL4 pathways [11,14]. This allows paracrine to influence multiple links of regeneration: activating proliferation and osteogenic differentiation of resident progenitor cells, stimulating neovascularization, and suppressing local inflammation through polarization of macrophages from the pro-inflammatory M1 phenotype to the anti-inflammatory M2 phenotype [15]. The main sources of therapeutic vesicles in bone engineering are traditionally various MSC populations, including bone marrow [16], adipose tissue [17], dental pulp [18], periodontal ligament [19], and umbilical cord MSCs [20].

Despite tissue-specific differences in their secretome, sEVs of native MSCs of different origins often demonstrate a lack of specific pro-osteogenic factors, which, combined with their rapid clearance from the defect area, limits their clinical efficacy in critical bone injuries. Therefore, in modern bioengineering, the focus has shifted to the use of modified sEVs obtained through various strategies, including physicochemical modification of donor cells, direct modification of isolated vesicles, and genetic modification of donor MSCs.

This review systematizes recent advances in the creation of modified MSC-derived EVs for bone tissue regeneration. The work also evaluates the potential of combining modified EVs with modern scaffold technologies and the prospects for their translation into clinical practice of bone tissue bioengineering.

This review was based on a narrative literature search of publications published between January 2010 and May 2026 in PubMed and Google Scholar. The search focused on modification strategies for MSC-derived extracellular vesicles in the context of bone-defect regeneration. Priority was given to original studies that reported a clearly defined MSC source and EV isolation procedure, an EV modification strategy, and experimental evidence of effects on the osteogenic, angiogenic, and/or immunomodulatory components of bone regeneration in relevant in vitro or in vivo models. For genetic-engineering approaches, the search specifically considered studies involving the delivery or overexpression of regulatory miRNAs and the osteogenic or angiogenic genes BMP2 and VEGFA in donor MSCs. Individual miRNAs, mRNAs, proteins, and signaling mediators are discussed only with direct citation of the corresponding original study. Studies were excluded from the mechanistic synthesis when the MSC source or EV isolation procedure was insufficiently described, when the reported cargo alteration was not linked to a relevant functional outcome, or when the study did not address bone regeneration or directly relevant osteogenic, angiogenic, or immunomodulatory processes.

## 2. Native MSC-Derived EVs

The study of the potential of MSC derivatives in bone engineering begins with the investigation of native EVs isolated from intact cultures. As a key component of the paracrine secretome of MSCs, native EVs are able to initiate a regenerative response in the damage zone by acting on resident osteoprogenitor cells, endothelial cells, and immune cells [21].

The most studied source of vesicles is traditionally bone marrow MSCs (BMSCs). In combination with polydopamine (PDA)-coated biomaterials, BMSC sEVs are able to recruit endogenous stem cells, stimulate their proliferation, and locally increase the expression of Runt-related transcription factor 2 (*Runx2*), collagen type I (*COL1*), and osteocalcin (*OCN*) genes associated with the early, middle, and late stages of osteogenic differentiation [16]. The osteogenic potential of BMSC EVs was also shown upon their release from a biocompatible polylactic acid (PLA)-based scaffold: EVs isolated from human BMSCs stimulate osteogenic differentiation, which is manifested by an increase in alkaline phosphatase (ALP) activity, increased expression of *Runx2* and structural protein genes (*OCN*, bone sialoprotein (*IBSP*)), as well as enhanced mineralization of the extracellular matrix. In addition, BMSC EVs are efficiently internalized by cells of the macrophage line RAW264.7. Upon local administration as part of a PLA scaffold, these vesicles exhibit pronounced immunomodulatory and antioxidant properties: in LPS-stimulated RAW264.7 macrophages, a decrease in reactive oxygen species levels and suppression of the expression of key pro-inflammatory cytokines such as interleukin-1β (IL-1β), inducible nitric oxide synthase (iNOS), interleukin-6 (IL-6), and tumor necrosis factor α (TNFα) are observed, which forms a favorable microenvironment in the defect area for tissue regeneration [22].

Dental MSC-derived sEVs also possess pronounced potential for bone regeneration. In particular, sEVs of periodontal ligament stem cells (PDLSCs) obtained from healthy patients are able to suppress hyperactivation of the canonical Wnt pathway, restoring the osteogenic potential of periodontal ligament cells taken from patients with periodontitis. The results were confirmed in an in vivo study on a model of experimental periodontitis in rats, where upon application of matrigel impregnated with sEVs of healthy PDLSCs, an increase in the volume of newly formed bone tissue in the alveolar process area and restoration of the osteogenic capacity of endogenous stem cells were observed [23]. The therapeutic efficacy of PDLSC sEVs in restoring alveolar bone defects in rats is enhanced upon their immobilization in biocompatible hydrogel matrices, which provides a pronounced positive effect on the proliferative capacity of BMSCs in vitro and significantly accelerates the rate of bone tissue regeneration in the damage area in vivo [19]. At the molecular level, sEVs isolated from PDLSCs substantially enhance osteoblast differentiation of target cells through activation of key osteogenesis markers, including ALP, BMP-2, OCN, and osteopontin (OPN) [24]. The combination of dental MSC EVs with angiogenic growth factors represents a promising strategy for simultaneous stimulation of angiogenesis and osteogenesis in bone tissue regeneration. Thus, the application of a composite system based on a thermosensitive PLGA-PEG-PLGA hydrogel with incorporated VEGF and chitosan microspheres containing dental pulp cell (DPSC) EVs in a model of critical calvarial defect in rats leads to a significant increase in the volume of newly formed bone tissue with simultaneous formation of a developed vascular network [18].

Adipose-derived MSC (ADSC) sEVs also possess pronounced therapeutic potential, exerting coupled osteoinductive, proangiogenic, and anti-inflammatory effects. In the study, it was demonstrated that a composite scaffold based on poly(lactic-co-glycolic acid) (PLGA) and Mg-GA MOF, functionalized with ADSC sEVs and providing prolonged release of sEVs, Mg^2+^, and gallic acid, possesses pronounced osteogenic and proangiogenic properties and promotes the formation of new vessels and acceleration of bone remodeling processes [25]. Impregnated into matrices based on gelatin sponge with PDA coating, ADSC sEVs stimulate BMSC proliferation in vitro, increase the expression of osteogenic markers (RUNX2, Osterix (OSX), OCN, OPN, COL1), ALP activity, and extracellular matrix mineralization, significantly accelerating regeneration in a rat femoral defect model [17]. It was also shown that sEVs applied to porous titanium scaffolds with hydroxyapatite coating promote bone regeneration, which was confirmed by a significant increase in ALP activity in in vitro experiments and a reliable improvement in the qualitative parameters of newly formed bone in a rabbit mandible defect model compared to scaffolds without sEVs [26].

Alternative sources are perinatal MSCs (umbilical vein or human umbilical cord (hucMSCs) and urine-derived stem cells (USCs)). sEVs obtained from hucMSCs, in combination with biomaterials in vitro, exert osteoinductive and proangiogenic effects, stimulating osteogenic differentiation of osteoprogenitor cells, as well as proliferation, migration, and vascular network formation by human umbilical vein endothelial cells (HUVECs), and also show increased regenerative capacity in vivo [20,27]. These effects may be associated with upregulation of HIF-1α and control of the expression of key osteogenic factors, in particular *BMP2* and *VEGF* [27]. In an in vitro study, the proangiogenic effect of hucMSCs sEVs was enhanced with increasing concentration. Under in vivo conditions, the combination of hucMSCs sEVs with 3D-printed scaffolds based on silk fibroin, COLI, and nanohydroxyapatite (nHA) promotes the restoration of alveolar bone defects through simultaneous stimulation of angiogenesis and osteogenesis [20]. In turn, USC sEVs encapsulated in a GelMA-HAMA/nHAP-based hydrogel increase Hypoxia-Inducible Factor 1 (HIF1A) expression in endothelial progenitor cell culture (EPCs), and also enhance the expression of osteogenic differentiation markers Runx2, OCN, and BMP-2, simultaneously affecting angiogenesis and osteogenesis under oxygen deficiency [28].

The biological activity of native MSC vesicles is largely mediated by their internal miRNA cargo. BMSCs EVs contain miR-590-3p, which directly suppresses the expression of the TGFBR1 receptor, which plays a negative role in the regulation of osteoinduction [29]. miR-181a plays a similar role [30], and vesicles enriched with miR-196a stimulate the expression of ALP, OCN, OPN, and RUNX2, and when administered as part of hydrogel carriers effectively stimulate bone formation in models with calvarial defects [31]. The angiogenic potential of native EVs is largely determined by exosomal miR-21, which suppresses the NOTCH1/DLL4 signaling pathway in recipient cells [32].

Despite the demonstrated efficacy, the native MSC secretome has internal limitations that hinder its widespread application. The composition of vesicles demonstrates heterogeneity: along with pro-osteogenic factors, intact EVs may contain regulators with the opposite vector of action. Thus, the expression of miR-885-5p exerts a negative regulatory influence on osteogenic activity induced by BMP-2 through suppression of the key factor Runx2 [33]. In addition, the natural concentration of therapeutically valuable molecules in native EVs is often insufficient for the regeneration of critical bone defects, and the vesicles themselves are subject to rapid washout. These limits predetermined the need for the development of improved approaches aimed at controlled enhancement of their osteoinductive properties by modifying EVs or donor cells.

## 3. EVs Derived from Physicochemically Modified MSCs

**Figure 1:**
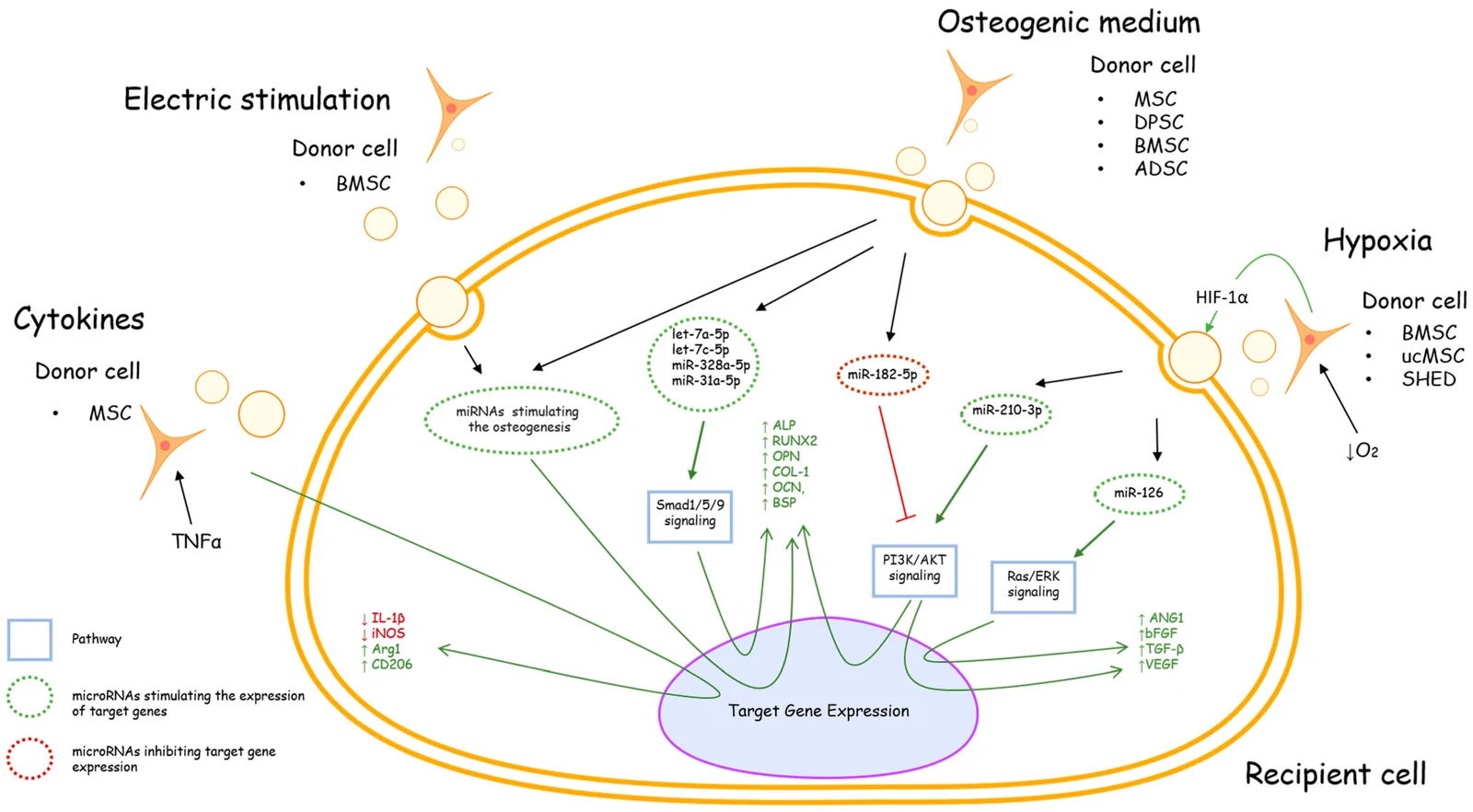
Effects of physicochemical modulation of donor cells on extracellular vesicle cargo and molecular pathways associated with osteogenesis, angiogenesis, and immunomodulation in recipient cells.

Currently, to enhance the regenerative potential of EVs, physicochemical methods such as preconditioning of donor cells in specific osteoinductive media supplemented with dexamethasone, β-glycerophosphate, and ascorbic acid [34], hypoxic preconditioning [35], mechanical stimulation, and electromagnetic stimulation [36] are used (Figure 1).

Often, pre-cultivation of MSCs in an osteogenic medium to specifically change the functional properties of secreted vesicles is used. This approach allows the molecular content of secreted EVs to be altered already at the stage of their biogenesis, thereby enhancing their osteogenic potential. Thus, experimental data demonstrate that sEVs from MSCs isolated after 10 and 15 days of differentiation possess more pronounced osteogenic potential than vesicles from undifferentiated cells. Transcriptomic analysis reveals that this was due to their unique molecular composition, characterized by increased content of pro-osteogenic miRNAs (Hsa-miR-146a-5p, Hsa-miR-503-5p, Hsa-miR-483-3p, Hsa-miR-129-5p) with a simultaneous decrease in the levels of miRNAs that suppress osteogenesis (Hsa-miR-32-5p, Hsa-miR-133a-3p, and Hsa-miR-204-5p) [37]. Similar changes in the miRNA profile were detected in sEVs isolated from DPSCs. In particular, incubation of cells in osteogenic medium induces the secretion of vesicles with increased expression of pro-osteogenic miRNAs Hsa-miR-29c-5p, Hsa-miR-378a-5p, Hsa-miR-10b-5p, and Hsa-miR-9-3p and reduced levels of miRNAs that negatively regulate osteogenesis, such as Hsa-miR-31-3p, Hsa-miR-221-3p, Hsa-miR-183-5p, and Hsa-miR-503-5p [38]. Moreover, the application of sEVs isolated from DPSCs after preliminary osteogenic differentiation as part of biocompatible polymeric PLGA-PEG-PLGA matrices in a rat bone defect model promotes controlled release of sEVs from the scaffold, ensuring recruitment of endogenous cells and stimulating bone tissue neogenesis in vivo [39]. Additional data show that preliminary osteogenic differentiation of BMSCs is accompanied by changes in the functional properties of secreted EVs. In an in vitro study, such vesicles enhance the osteogenic differentiation of BMSCs and stimulate the formation of vessel-like structures by HUVECs. In addition, the obtained MSC EVs exert an immunomodulatory effect on RAW264.7 macrophages. Analysis of the molecular content of the vesicles and subsequent investigation of the mechanism of action reveal the involvement of miR-18a-5p in the regulation of the p53 signaling pathway, which links the change in the molecular composition of exosomes to their immunomodulatory effect [40]. The biological efficacy of sEVs from pre-differentiated MSCs is confirmed by comparative studies: compared with sEVs obtained before the onset of osteogenic induction, vesicles collected on day seven of osteoinduction are characterized by an increased level of pro-osteogenic miRNAs and a decreased level of anti-osteogenic miRNAs, in particular miRNA-182-5p. These changes are associated with enhanced osteogenic differentiation of BMSCs and activation of the IGF1R/PI3K/Akt osteogenic signaling pathway [41]. The osteoinductive properties of sEVs may also depend on the origin of the donor cells. In comparative tests, sEVs from BMSCs previously exposed to osteoinductive medium demonstrated a stronger effect on osteogenesis in vitro compared with sEVs from ADSCs. BMSC exosomes induced increased expression of a broad range of osteogenesis markers—Col1, Runx2, IBSP, OCN, Bmpr1a, and Bmpr2—whereas exosomes from ADSCs affected a limited pool of genes, namely IBSP, Bmpr1a, and Bmpr2 [34]. The high efficacy of BMSC sEVs is due to the combined action of the miRNAs they contain (let-7a-5p, let-7c-5p, miR-328a-5p, and miR-31a-5p), which activate the Smad1/5/9 phosphorylation cascade in recipient cells. Immobilization of BMSC sEVs on hierarchically structured bioactive glass scaffolds ensures their biological activity, prolonged release, and substantially enhances the osteogenic potential of the scaffold, stimulating bone regeneration in a rat calvarial defect model [34].

In addition to cultivation in osteogenic medium, cytokine conditioning of donor cells allows the functional properties of secreted vesicles to be deliberately altered. Thus, preliminary stimulation of MSCs with TNFα qualitatively changes the nature of the paracrine response: the resulting sEVs acquire the ability to effectively suppress inflammation by inducing polarization of RAW264.7 macrophages from the pro-inflammatory M1 phenotype to the regenerative M2 phenotype, which is confirmed by decreased expression of IL-1β, iNOS, and pronounced expression of arginase-1 (Arg1) and mannose receptor (CD206) [42].

The most important vector of physical modification of the paracrine activity of MSCs is hypoxic preconditioning, aimed at stimulating coupled angio-osteogenesis. Cultivation of BMSCs under hypoxic conditions leads to the secretion of modified vesicles (hypo-sEVs) that induce migration and proliferation of HUVECs much more effectively than vesicles obtained under normoxic conditions. miRNA analysis and subsequent experiments reveal the key role of miR-210-3p, whose content in hypo-sEVs increases due to HIF-1α-dependent regulation under hypoxia, which activates the proangiogenic PI3K/AKT pathway in target cells [35]. Regardless of the method of enhancing the angiogenic signal, its functional significance for bone regeneration is shown in the work of Takeuchi et al. (2019): sEVs from a conditioned medium of BMSCs enhanced cell migration, expression of angiogenic (including VEGF) and osteogenic genes, and early bone formation in a calvarial defect model, whereas neutralization of VEGF with a specific antibody substantially attenuates these effects [43]. A similar effect of hypoxia was detected for sEVs obtained from hucMSCs. Studies reveal that hypoxic sEVs enriched with miR-126 stimulate angiogenic and proliferative activity, as well as the migratory potential of cells, through activation of the Ras/Erk signaling pathway. Importantly, the transcription factor HIF-1α played a key role in regulating miR-126 expression in MSCs and its subsequent accumulation in secreted sEVs, which emphasizes its central importance in forming the proangiogenic content of sEVs during hypoxic preconditioning [44]. In a critical calvarial defect model, the application of hypoxic sEVs leads to a substantial enhancement of not only neovascularization but also bone regeneration in general [35]. Comprehensive proteomic analysis showed that hypoxic preconditioning enhances sEV secretion by human stem cells from exfoliated deciduous teeth (SHEDs), triggering the VEGF signaling pathway. Local delivery of such hypoxic sEVs using polydopamine-coated porous PLGA microspheres ensures their controlled release over 21 days, leading to pronounced enhancement of both neovascularization and osteogenesis in models of critical calvarial bone defects in rats [45].

To improve the regenerative properties of MSC sEVs, physical methods are also used, for example, electrical stimulation of BMSCs, which enriches sEVs with oxidative phosphorylation proteins to activate the PI3K-Akt and MAPK pathways in target cells [36]. The application of such sEVs as part of hydrogels substantially accelerates the early stages of bone tissue regeneration in femoral defect models.

Thus, current studies show that modifications of EVs using physicochemical approaches significantly enhance their regenerative properties. Methods of physicochemical influence on MSCs allow for comprehensive optimization of the therapeutic profile of EVs, simultaneously affecting osteogenesis, angiogenesis, and the local immune response. However, this strategy is limited by the natural metabolic capabilities of the donor cell and is associated with difficulties in standardizing the composition of vesicles. To overcome these limitations and ensure targeted controlled loading of osteogenic factors, bone tissue bioengineering turns to an alternative approach—direct physicochemical modification of already isolated and purified EVs, which is considered in the next section.

## 4. Modified MSC-Derived EVs

Direct modification of isolated and purified MSC EVs represents an alternative strategy for controlling the regenerative potential of EVs. Depending on the task, this approach can be conventionally divided into two main directions: altering the composition of EVs to deliver additional therapeutic factors and modifying their surface for immobilization on biomaterials, local retention, and prolonged release in the bone defect area. The first group includes electroporation and co-incubation, used for loading plasmids, mRNA, miRNA, siRNA, and other functional molecules, as well as fusion of EVs with liposomes containing a therapeutic cargo. The second direction includes surface engineering modification of EVs by attaching peptides, proteins, or other ligands that ensure their binding to components of the extracellular matrix or biomaterials, thereby enhancing local retention and prolonged release.

One approach to altering the contents of isolated EVs is co-incubation of native vesicles with specific regulatory molecules. Thus, native BMSC exosomes were modified with a cholesterol-conjugated inhibitor of miR-182-5p. The resulting constructs retained high stability in vitro and in vivo, and vesicles modified with the miR-182-5p inhibitor enhanced osteogenic differentiation of BMSCs and stimulated new bone tissue formation compared with unmodified vesicles [41].

To overcome the problem of low natural EVs yield, the method of sequential mechanical extrusion of cell suspensions through titanium filters with a gradient decrease in pore size is used. The homogeneous exosome-mimetic particles (EMs) obtained in this way contained a significantly higher amount of protein and retained the ability to be internalized by recipient cells [46,47]. Encapsulation of a plasmid construct expressing VEGF into the obtained EMs by electroporation exerted a pronounced proangiogenic effect in HUVECs [46]. Modified vesicles did not significantly affect the expression of the osteogenic marker genes *Runx2* and *Ocn*, but significantly increased the expression of *Alp* and *Col1a1* in rat BMSCs compared with the unmodified group, and also enhanced the efficiency of bone defect regeneration in rat radius defects [48].

In addition to introducing genetic constructs, physicochemical modification of the EV surface is also used to increase its affinity for implantation materials, which prevents rapid washout of the preparation from the defect area. For this purpose, bifunctional fusion proteins containing a titanium-binding domain and the CP05 peptide, which has high affinity for the conserved marker CD63 on the exosomal membrane, are used. Co-incubation of native vesicles with such hybrid constructs ensures their reliable retention on the surface of porous titanium scaffolds, which enhances the osteoinductive properties of the matrix in vitro and leads to local accumulation of sEVs in the implantation area in vivo [49]. Of no less interest is the use of high-affinity systems for polymeric carriers; thus, VEGF-containing EMs specifically bound to nanofibrous matrices of chitosan/polylactide obtained by electrospinning, through biotin-avidin interaction, ensured prolonged release of EMs directly in the bone defect area [46].

Despite the variability of approaches, technologies for direct modification of isolated EVs have a number of limitations that potentially hinder their translation into clinical practice. Physicochemical manipulations are associated with the risk of disrupting the integrity of the lipid bilayer of vesicles and altering their biological properties [50], and the loading efficiency and preservation of the functional activity of the vesicle contents depend on the method used and the characteristics of the molecule itself. In particular, during electroporation, the efficiency of loading exogenous DNA decreases with increasing size of the DNA construct, and functional delivery of large DNA molecules may remain limited [51]. In addition, these protocols require additional labor-intensive purification steps from cellular metabolites and are difficult to scale up industrially [51,52]. All of the above predetermined the transition of bone tissue bioengineering to the most advanced strategy—programming the composition and topology of vesicles directly at the DNA level of donor cells, which is considered in the next section.

## 5. EVs Derived from Genetically Modified MSCs

**Figure 2:**
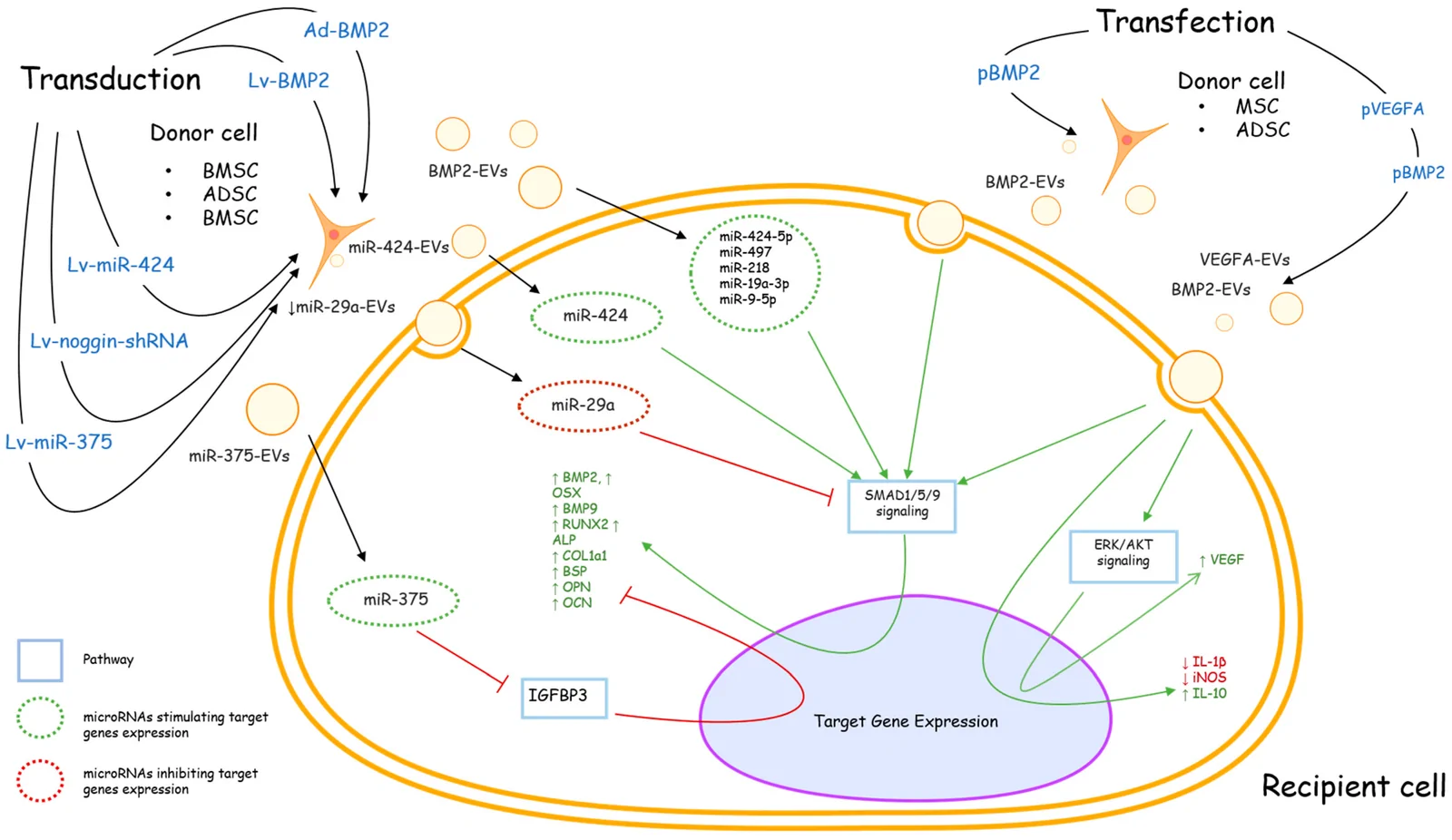
Effects of extracellular vesicles derived from genetically modified mesenchymal stromal cells, carrying therapeutic mRNAs and regulatory miRNAs, on molecular pathways in recipient cells.

The most technologically advanced direction for enhancing the therapeutic efficacy of MSC-derived EVs in bone tissue engineering is the genetic modification of donor cells. Unlike physicochemical methods, this strategy allows the molecular content of secreted EVs to be altered already at the stage of their biogenesis. For the delivery of target nucleic acids into MSCs, viral transduction methods (e.g., using lentiviral or adenoviral vectors) as well as non-viral transfection (including liposomal transfection and nanoelectroporation) are successfully used. Depending on the chosen genetic construct, targeted reprogramming of donor MSCs can lead to enrichment of EVs with therapeutically relevant mRNAs and regulatory miRNAs, which, after delivery to target cells, are able to increase the expression of genes and proteins associated with osteogenic differentiation, activate osteogenic signaling pathways, or suppress their negative regulators. Moreover, genetic modification of donor MSCs can alter not only the content of the target mRNA or miRNA but also other components of the molecular cargo of the vesicles. As a result, interconnected signaling cascades involving several molecular links are activated in recipient cells, which can provide a complex and potentially more balanced regenerative effect (Figure 2).

One of the most studied variants of genetic reprogramming is the enhancement of the osteogenic potential of EVs through overexpression of BMP-2 in donor MSCs [53]. Constitutive expression of *BMP2* in donor BMSCs, achieved by stable lentiviral transduction, led to the production of functionally enhanced exosomes (FEEs). Exposure of such FEEs to intact BMSCs in vitro activated the pro-osteogenic SMAD1/5/8 cascade and increased the expression of differentiation factors *BMP2*, *RUNX2*, *OSX*, and *BMP9*. Transcriptomic analysis of the contents of FEEs revealed that this effect is due to an increase in the levels of five miRNAs—miR-424-5p, miR-497, miR-218, miR-19a-3p, and miR-9-5p—complementary to the osteogenesis inhibitors SMAD7 and SMURF1. In target cells, suppression of SMURF1 was accompanied by activation of the canonical BMP/Smad signaling pathway, which linked the altered miRNA levels in the vesicle cargo to enhanced osteogenic response. Under in vivo conditions in a rat calvarial defect model, administration of functionalized vesicles as part of a collagen sponge provided pronounced bone tissue regeneration without signs of ectopic bone formation or fatty infiltration of the matrix [53]. A similar approach using adenoviral transduction of donor cells allowed the BMP-2 mRNA content in sEVs to be increased approximately 3-fold. The resulting vesicles induced an increase in ALP activity, phosphorylation of SMAD1/5/8, and enhanced expression of the main transcription factors *RUNX2*, *Alpl*, and *Col1a1*. Local incorporation of such vesicles into an injectable collagen hydrogel ensured their release and increased the bone volume fraction in the femoral defect area by more than 3-fold [54].

A similar approach to enhancing the BMP-2-dependent effect was implemented using liposomal transfection of donor MSCs with a plasmid carrying the *BMP2* gene. sEVs secreted by genetically modified cells (MSC-BMP2-Exo) demonstrated more pronounced osteogenic activity toward acceptor cells compared with native sEVs. Analysis of mRNA expression in the enriched MSC-BMP2-Exo showed a significant increase in the content of not only *BMP2* but also other transcripts, including *Runx2*, *OSX*, *ALP*, *BSP*, and *OPN*, relative to native sEVs. The therapeutic efficacy of these sEVs was confirmed in vivo in a mouse model of a critical defect of the lateral epicondyle of the distal femur. On day 15 after implantation, a significant number of Smad1/5/8-positive cells were observed in the damage area in the group receiving MSC-BMP2-Exo [55].

A more complex variant of genetic programming involves the simultaneous enrichment of EVs with several therapeutically relevant mRNAs. In the study, using nanoelectroporation, mRNA-encoding BMP-2 and VEGF were delivered into human ADSCs with the aim of simultaneously stimulating osteogenesis and angiogenesis. The presence of BMP-2 and VEGF mRNAs was confirmed in the obtained vesicles. Under in vitro conditions, co-incubation with the obtained exosomes induced a sharp increase in the expression of osteogenic factors in recipient cells and activated the pro-osteogenic BMP-2/Smad cascade. Under in vivo conditions, the obtained therapeutic sEVs were incorporated into an injectable PEGS-A hydrogel and administered to the area of a critical femoral defect in rats. Compared with native sEVs, modified vesicles enriched with BMP-2 and VEGF-A mRNAs provided more pronounced formation of new bone tissue and vascular network in the defect area, which indicated enhancement of the coupled processes of angiogenesis and osteogenesis. At the same time, the authors showed that genetic programming of donor cells was accompanied by changes in a number of endogenous miRNAs in the vesicle cargo. This indicates the possibility of broader reprogramming of the molecular content of EVs upon alteration of the genetic program of donor cells, as a result of which the observed therapeutic effect may be determined by the combined action of introduced and endogenous components [56].

In addition to introducing genes encoding osteogenic factors, genetic programming of donor MSCs can be aimed at suppressing negative regulators of osteogenesis and, consequently, activating the corresponding signaling pathways in recipient cells. Thus, the creation of a stable line of human ADSCs with overexpression of miR-375 by lentiviral transduction led to an increase in the miR-375 content in secreted sEVs. Upon entering target cells, miR-375 suppressed the expression of Insulin-like Growth Factor Binding Protein-3 (IGFBP3)—a protein inhibiting osteogenesis—which contributed to enhanced osteogenic differentiation. Local delivery of such vesicles as part of hydrogels based on hydroxyapatite and thiol-modified hyaluronic acid and heparin provided a significant acceleration of cranial defect regeneration in rats compared with control sEVs [57]. A similar strategy was used for targeted enrichment of EVs with miR-424. Lentiviral transduction of BMSCs with a construct ensuring EV-specific incorporation of miR-424 allowed for the production of EVs enriched with the target miRNA. After uptake by recipient cells, such EVs activated the endogenous BMP2/SMAD1/5/8 signaling cascade, which was confirmed by increased phosphorylation of SMAD1/5/8, as well as enhanced expression of osteoinductive genes and matrix mineralization. The proposed mechanism of this effect is attenuation of the miR-424-dependent activity of negative regulators of BMP-2 signaling, including SMAD7 and SMURF1. Such enriched EVs as part of a collagen carrier significantly accelerated the regeneration of rat calvarial defects compared with the native secretome [58]. Another variant consists of suppressing an endogenous antagonist of BMP signaling directly in donor cells. In the work, Noggin expression in hMSCs was reduced by lentiviral transduction of shRNA followed by selection of stably transduced cells. EMs obtained from such cells contained an increased amount of noggin siRNA, a reduced level of Noggin protein, and a decreased content of miR-29a. The reduction in miR-29a attenuated the suppression of expression of the BMP type I receptor BMPR1A, which contributed to enhanced signal transmission through the BMP/SMAD cascade and increased osteoinductive activity of EMs. Such EMs possessed pronounced osteoinductive potential already at a concentration of 5 µg/mL, significantly surpassing EMs from unmodified cells. Moreover, this effect was strictly dose-dependent and reached its maximum at a concentration of 30 µg/mL. In combination with an injectable hydrogel based on methacrylated glycol chitosan, EMs stimulated regeneration of a critical calvarial defect in mice [47].

Thus, the considered data indicate that the functional enhancement of EVs is determined not only by the presence of the introduced genetic construct but also by the rearrangement of their molecular content. It is also capable of altering the interaction of vesicles with recipient cells and the subsequent activation of intracellular signaling pathways. Importantly, genetic engineering of donor MSCs may introduce changes extending beyond enrichment with the intended therapeutic cargo. Viral transduction, prolonged selection of transduced cell populations, and ectopic transgene expression may affect donor-cell proliferation, stress responses, differentiation state, inflammatory signaling, and secretory activity. These changes can, in turn, influence the abundance and composition of EV-associated RNAs, proteins, lipids, and surface molecules independently of the target construct. Consequently, improved osteogenic, angiogenic, or immunomodulatory outcomes in preclinical models cannot always be attributed exclusively to the introduced miRNA or mRNA.

For the application of modified EVs in bone-defect conditions, an important addition is provided by matrix systems that allow the vesicles to be localized in the damage area and to control the time of their presence in the regenerating tissue. The combination of genetic programming of donor cells with local matrix delivery creates prerequisites for the development of more controlled EV preparations with a defined regenerative profile and expands the possibilities for overcoming the limitations of the native secretome. Nevertheless, successful clinical translation of this technology directly depends on the standardization of large-scale production methods, rigorous quality control, and comprehensive preclinical safety evaluation, as discussed in the next section.

## 6. Advantages and Limitations of Engineered MSC-Derived EVs

The transition from the use of native MSC-derived products to strategies for their targeted engineering has improved the effectiveness of bone tissue regeneration. Modified MSC-derived EVs offer several advantages over traditional cell therapy. As cell-free biologics, they are characterized by minimal immunogenicity and high biocompatibility, which leads to a minimal risk of immune rejection, and also demonstrates a complete absence of risks of malignant transformation, ectopic bone formation or vascular embolism, with strong regulatory and ethical restrictions [59,60,61]. The lipid bilayer of EVs provides reliable protection of their molecular cargo from degradation and facilitates its delivery to target cells [59,62]. A key determinant of the functional profile of engineered EVs is the modulation of their molecular cargo. Genetic modification or preliminary exposure to donor cells can change the content of regulatory RNAs in secreted vesicles, thereby influencing signaling pathways and gene expression in recipient cells. The data presented in Table 1 provide a link between changes in EV cargo and the direction of the biological response, including the regulation of osteogenic, angiogenic, and immunomodulatory processes.

However, the widespread implementation of modified MSC vesicles in clinical orthopedic practice remains constrained by substantial technological and biological limitations. The main economic and production barriers are the extremely low yield of small vesicles per unit of cell mass, which requires the cultivation of huge volumes of MSCs and slightly increases the final cost of therapy [63]. Despite the emergence of advanced electrochemical microfluidic devices conjugated with antibodies to CD9, CD63, and CD81 markers for selective adsorption of EVs under voltage, the possibility of scaling up such purification systems remains limited [64]. Additional obstacles arise from the difficulty of standardizing batch composition, which varies depending on MSC culture conditions [65], as well as stringent requirements for transportation and the limited availability of long-term clinical safety data [66,67]. A significant biological limitation is the rapid elimination of EVs from circulation [68]. This process is caused by the expression of phosphatidylserine on the vesicle membrane, which leads to their recognition by resident macrophages and subsequent rapid phagocytosis [69,70].

This variability reflects the intrinsic heterogeneity of MSC-derived EV preparations. EV cargo depends not only on the tissue source and donor of MSCs but also on clonal diversity, cellular senescence, passage number, seeding density, culture medium, oxygen tension, conditioning protocol, harvest time, and isolation procedure [71]. Consequently, a bulk EV preparation represents a distribution of vesicle subpopulations rather than a uniform therapeutic entity. Current analytical approaches, including nanoparticle tracking analysis, electron microscopy, immunoblotting, flow cytometry, proteomics, and RNA sequencing, provide complementary information on particle size, concentration, morphology, selected markers, and average cargo composition at the population level. However, they cannot comprehensively determine the complete RNA, protein, lipid, and surface-molecule profile of every individual EV [9,72]. Single-EV technologies, such as nanoscale flow cytometry, single-particle interferometric imaging, super-resolution microscopy, Raman-based approaches, and microfluidic platforms, are beginning to resolve selected physical features and limited panels of surface markers or nucleic acids at the single-vesicle level [72]. Nevertheless, these methods remain constrained by limited analytical sensitivity, incomplete multiplexing capacity, variable detection thresholds, challenges in distinguishing EVs from non-vesicular particles, and the absence of standardized workflows [73]. Thus, they are not yet suitable for comprehensive, routine cargo profiling of individual MSC-derived EVs or for batch-release testing. At present, robust characterization requires a combination of orthogonal population-level assays, predefined critical quality attributes, standardized donor cell culture and passage conditions, controlled collection and isolation protocols, and validated potency assays.

Given these analytical and manufacturing constraints, from a clinical translation perspective, choosing an EV engineering strategy requires balancing functional gain and process complexity, batch reproducibility, quality-control requirements, and regulatory burden. Cell pre-conditioning, direct modification of isolated EVs, and genetic engineering of donor cells differ substantially in their manufacturing workflows and in the level of control over the final product. A comparative assessment of these approaches is therefore essential for identifying strategies that are not only biologically effective but also compatible with scalable and reproducible production (Table 2).

Importantly, no single engineering strategy is universally optimal for clinical translation. Pre-conditioning offers the simplest and potentially most cost-effective upstream intervention, but its clinical utility depends on rigorous standardization of culture conditions and demonstration of stable batch-to-batch potency. Direct EV modification provides greater control over cargo loading, surface properties, and local retention; however, it adds downstream processing steps and requires verification of EV integrity, removal of unbound cargo or reagents, and preservation of biological activity. Genetic engineering of donor cells enables sustained production of EVs enriched with defined therapeutic cargo, but it entails the highest development cost, the most complex quality control framework, and the greatest regulatory burden, particularly when integrating viral vectors are used. Therefore, the translational suitability of each approach should be assessed not only by its regenerative efficacy but also by the feasibility of GMP-compatible manufacturing, definition of strategy-specific critical quality attributes, validated potency assays, lot-to-lot reproducibility, product stability, and long-term safety.

Targeted integration of modified MSC-derived EVs with advanced biomimetic carriers and “smart” hydrogels can enhance local vesicle retention and ensure their controlled release in the bone defect area. Although most data on the application of EV platforms for bone tissue regeneration remain preclinical, this direction has already moved to early clinical trials. In particular, a phase I/II trial NCT05520125 has been registered, which evaluates the use of MSC-enriched EVs in patients with segmental defects and bone loss. In addition, a phase I trial NCT05060107 is dedicated to assessing the safety and efficacy of a single intra-articular injection of MSC-derived sEVs CelliStem^®^OA-sEV in moderate knee osteoarthritis. However, the current recruitment status of both registered studies is listed as “Unknown” in the ClinicalTrials.gov database, and no study results have been posted to date. Thus, exosome and extracellular vesicle technologies have already moved to clinical evaluation for musculoskeletal diseases and injuries.

## 7. Conclusions

The approaches to MSC EVs engineering discussed above demonstrate the potential for targeted modification of their molecular content and functional properties to regulate osteogenic, angiogenic, and immunomodulatory responses. Furthermore, the effectiveness of vesicles can be determined not only by the incorporation of target genetic material but also by the accompanying rearrangement of their molecular cargo, highlighting the need for a comprehensive characterization of the composition and mechanisms of action of modified EVs. For their application in the treatment of bone defects, matrix-based systems are of particular importance, as they can localize vesicles to the site of injury and enable their controlled release. The combination of these approaches provides the basis for the development of EV drugs with more predictable functional properties and controlled local delivery. At the same time, further development in this area requires standardization of vesicle production and characterization methods, optimization of their production and storage conditions, as well as a more detailed study of the relationship between cargo composition, the physicochemical characteristics of EVs, and their therapeutic effect.

## Figures and Tables

**Table 1:** Regulatory RNA content in MSC-derived EVs.

Modifications	Donor Stem Cells	Isolation Method	Delivered Cargo	Target Cells	Affected Genes or Signaling Pathways	Therapeutic Effects	Resources
Native Cells	BMSC	Centrifugation (300× *g*, 2000× *g*, and 12,000× *g*) and filtered to remove particles > 200 nm.	Upregulating miR-590-3p	Human osteoblasts (hFOB1.19)	Downregulating TGFBR1	Promotion of osteogenic differentiation	[29]
	BMSC	Differential ultracentrifugation	Upregulating miR-885-5p	BMSCs	Downregulating Wnt5a and attenuating BMP2-induced RUNX2 expression	Inhibition of osteogenic differentiation	[33]
	BMSC	Differential ultracentrifugation	Upregulating miR-196a	Human osteoblasts (hFOB 1.19)	Not assessed	Promotion of osteoblast differentiation	[31]
	umbilical MSC	Differential ultracentrifugation	Upregulating miR-21	endothelial progenitor cells (EPCs)	Upregulating NOTCH1/DLL4 pathway	Promotion of angiogenesis	[22]
**Physicochemical Modifications of MSCs**							
Osteogenic induction medium	BMSC	Polymer-based precipitation	Upregulating Hsa-miR-146a-5p, Hsa-miR-503-5p, Hsa-miR-483-3p, Hsa-miR-129-5p	hMSCs	Activating PI3K/Akt and MAPK	Promotion of osteogenic differentiation	[37]
	hDPSC	Insufficiently described	Upregulating miR-29c-5p Hsa-miR-378a-5p, Hsa-miR-10b-5p and Hsa-miR-9–3p	hDPSCs	Activating MAPK and AMPK	Promotion of osteogenic differentiation	[38]
	hBMSCs	Insufficiently described	Downregulating miR-18a-5p	Raw264.7	Activating p53	Promotion of M2 macrophage polarization	[40]
	hBMSCs	Differential ultracentrifugation	Upregulating let-7a-5p, let-7c-5p, miR-328a-5p, miR-31a-5p	rBMSCs	Activating Smad1/5/9 phosphorylation	Promotion of osteogenic differentiation	[32]
Hypoxia	hBMSCs	Differential ultracentrifugation	Upregulating miR-210-3p	HUVECs	Downregulating EFNA3 gene expression and enhancing the phosphorylation of PI3K/AKT pathway	Promotion of angiogenesis	[35]
	HucMSCs	Ultrafiltration plus ultracentrifugation	Upregulating miR-126	HUVECs	Activating SPRED1/Ras/Erk signaling pathway	Promotion of endothelial cell proliferation, angiogenesis and migration	[44]
**Modifications of exosomes**							
Coincubation	AB-BMSCs	Differential ultracentrifugation	Down regulating miR-182-5p	BMSCs	Activating PI3K/Akt signaling pathway	Promotion of osteogenic differentiation	[41]
**Genetic engineering of donor cells**							
Lentiviral transduction	hBMSCs	Polymer-based precipitation	Upregulating miR-424-5p, miR-497, miR-218, miR-19a-3p, miR-9-5p	hBMSCs	SMURF1 downregulation was accompanied by activation of canonical BMP/Smad signaling pathway	Promotion of osteogenic differentiation	[53]
	hADSC	Differential ultracentrifugation	Upregulating miR-375	hBMSCs	Downregulating expression of IGFBP	Promotion of osteogenic differentiation	[57]
	hBMSC	Polymer-based precipitation	Upregulating miR-424	HMSCs	Activating SMAD 1/5/8	Promotion of osteogenic differentiation	[58]

The table is not intended as an exhaustive quantitative catalog of all miRNAs detected in MSC-derived EVs; it summarizes regulatory RNAs reported in original studies together with a relevant functional outcome.

**Table 2:** Clinical translation considerations for engineering MSC-derived EVs.

Strategy	Cell Pre-Conditioning	Direct EV Modification	Genetic Engineering of Donor Cells
**Main advantages**	Relatively simple upstream intervention; does not require direct manipulation of isolated EVs; can be integrated into the donor cell culture process.	Allows post-isolation loading with defined cargo and/or surface functionalization; can support local retention or targeting without permanent donor cell manipulation.	Enables sustained and potentially controlled production of EVs enriched with selected RNA or protein cargo; may be compatible with stable producer cell platforms and continuous production.
**Manufacturing cost and scalability**	Generally the lowest additional cost; potentially compatible with closed bioreactor systems, but yield and cargo composition may depend strongly on the conditioning protocol.	Additional processing, purification, and analytical steps increase cost; scalability may be limited by loading efficiency and losses during post-processing.	High initial development and validation costs; stable engineered cell lines may support scalable production, but vector construction, cell banking, and extensive comparability testing are required.
**Quality control challenges**	Requires strict control of oxygen tension, duration, medium composition, cytokine dose, cell passage, and donor cell state; potency may vary between batches.	Must verify loading efficiency, free-cargo removal, EV integrity, particle recovery, aggregation, size distribution, and retained bioactivity after manipulation.	Requires characterization of donor cell stability, transgene expression, off-target changes in EV cargo, particle yield, potency, and vector-related residuals; vector-only controls are essential.
**Regulatory considerations**	Comparatively lower regulatory complexity because no exogenous cargo or genetic construct is introduced; nevertheless, protocol-specific reproducibility, identity, purity, potency, and safety must be demonstrated.	Intermediate regulatory complexity; the modified EV preparation must be characterized as a combined product, including potential effects of loading reagents, residual free cargo, and altered biodistribution.	Highest regulatory burden, particularly for integrating viral vectors; requires extensive evaluation of genomic stability, residual vector-related contaminants, biodistribution, immunogenicity, tumorigenicity, and long-term safety.

## Data Availability

No new data were created or analyzed in this study. Data sharing is not applicable to this article.

## Abbreviations

The following abbreviations are used in this manuscript:

ADSCs	Adipose-derived mesenchymal stem cells
ALP	Alkaline phosphatase
Arg1	Arginase-1
BMSCs	Bone marrow mesenchymal stem cells
BMP-2	Bone morphogenetic protein 2
BMPR1A	Bone morphogenetic protein receptor type IA
BSP	Bone sialoprotein
COL1	Collagen type I
DPSCs	Dental pulp stem cells
EMs	Exosome mimetics
EPCs	Endothelial progenitor cells
EVs	Extracellular vesicles
FEEs	Functionally enhanced exosomes
HIF-1α	Hypoxia-inducible factor 1α
hucMSCs	Human umbilical cord mesenchymal stem cells
HUVECs	Human umbilical vein endothelial cells
IGFBP3	Insulin-like growth factor binding protein 3
IL-1β	Interleukin-1β
IL-6	Interleukin-6
iNOS	Inducible nitric oxide synthase
MSCs	Mesenchymal stem cells
OCN	Osteocalcin
OPN	Osteopontin
OSX	Osterix
PDA	Polydopamine
PDLSCs	Periodontal ligament stem cells
PEG	Polyethylene glycol
PLA	Polylactic acid
PLGA	Poly(lactic-co-glycolic acid)
RUNX2	Runt-related transcription factor 2
sEVs	Small extracellular vesicles
SHEDs	Stem cells from human exfoliated deciduous teeth
TGF-β	Transforming growth factor β
TGFBR1	Transforming growth factor beta receptor 1
TNF-α	Tumor necrosis factor α
USCs	Urine-derived stem cells
VEGF	Vascular endothelial growth factor
